# Dynamics and Diversity of Microbial Community Succession During the Solid-State Fermentation Process of Fuzhuan Brick Sea Buckthorn Leaf Tea

**DOI:** 10.3390/foods15101727

**Published:** 2026-05-14

**Authors:** Yulu Wang, Jialu Ao, Qiankun Guo, Zhiyong Xie, Xia Fan, Yi Sun, Zhipeng Wang, Jinghong Wei, Xiaoxiong Zeng

**Affiliations:** College of Food Science and Technology, Nanjing Agricultural University, Nanjing 210095, China

**Keywords:** sea buckthorn (*Hippophae rhamnoides* L.), fermentation, Fuzhuan brick sea buckthorn leaf tea, Illumina NovaSeq 6000 sequencing, microbial community

## Abstract

Sea buckthorn (*Hippophae rhamnoides* L.) leaves are rich in nutrients and bioactive constituents, with great potential for fermented tea development. It has been demonstrated that Fuzhuan brick tea processing can improve sea buckthorn leaf tea flavor, but the underlying microbial succession remains unexplored. Therefore, we characterized the dynamic succession and interrelationships of bacterial and fungal communities via Illumina NovaSeq 6000 sequencing. β-diversity analysis revealed successive shifts in microbial community structure, with fungal communities changing mainly in the early stage and bacterial communities varying more in the late stage of fermentation. The relative abundance of *Pseudomonas*, a genus frequently associated with flavor formation and tea quality, increased steadily. Fungal taxonomic analysis revealed that the genus *Aspergillus*, particularly the species *Aspergillus chevalieri*, remained dominant throughout the fermentation process. Linear discriminant analysis effect size indicated an enrichment of microbial taxa typical of fermentation, accompanied by a relative reduction in putative opportunistic microbes. Additionally, *Aspergillus* exhibited significant negative correlations with five key differentially abundant bacterial genera. Interestingly, microbial co-occurrence networks suggested an overall tendency toward coexistence rather than mutual exclusion between the bacterial and fungal communities. This work provides a theoretical foundation for the development of novel fermented sea buckthorn leaf tea products.

## 1. Introduction

Fuzhuan brick tea (FBT) is a unique post-fermented tea in the dark tea category and is one of the six major types of Chinese tea. It is predominantly produced in Shaanxi and Hunan Provinces of China [1], from the leaves and buds of the tea plant (*Camellia sinensis* L.) through a sequence of sophisticated processing steps, such as pre-fermentation, blending, steaming, pressing, shaping, and an extended period of natural fermentation [2]. Unlike other fermented teas, such as Oolong and black teas, which primarily rely on enzymatic oxidation induced by endogenous enzymes in tea leaves, the post-fermentation of FBT depends on auto-oxidation and enzymatic oxidation induced by microorganisms in tea leaves [3]. A distinctive characteristic of FBT is the formation of “golden flowers” on its surface, which refers to the golden cleistothecia primarily produced by the fungus *Aspergillus cristatus* [4]. The dominant fungi in FBT can decompose macromolecules (e.g., starch and cellulose) and catalyze the oxidation and condensation of polyphenols, generating compounds such as thearubigins and theabrownins [5]. Due to its unique microbial characteristics, FBT not only possesses distinctive flavor profiles but also exhibits various health benefits, including anti-obesity, blood glucose reduction, and gut microbiota regulation effects, making it a topic of extensive research [6].

Sea buckthorn (*Hippophae rhamnoides* L.), also known as sea berry, belongs to the *Elaeagnaceae* family and is widely distributed in China, Mongolia, Russia, and Northern Europe [7]. Sea buckthorn is utilized for desertification control, afforestation and forest protection, soil environment improvement, and erosion prevention, and has significant economic and environmental value. Moreover, sea buckthorn is widely recognized as a natural medicine and has long been classified as a plant with dual medicinal and food properties by the National Health Commission of the People’s Republic of China [8]. Sea buckthorn, particularly its fruit, is rich in phenolic compounds, polysaccharides, vitamins, carotenoids, and unsaturated fatty acids, exhibiting various biological effects, including antioxidant, anti-inflammatory, lipid-lowering, and hypoglycemic activities, as well as the ability to regulate gut microbiota, protect the liver, boost immunity, and brighten the skin [9,10,11,12,13,14,15]. Notably, sea buckthorn leaf extracts have been reported to exhibit stronger antioxidant activity than fruit extracts [16]. However, current research primarily focuses on the fruit, seed extracts, and processed products, with insufficient investigation of sea buckthorn leaves, resulting in the underutilization of sea buckthorn resources. In fact, China has the richest natural sea buckthorn germplasm resources in the world and the largest area under artificial cultivation with a total planting area of 2.1 million hectares [17]. Therefore, sea buckthorn leaves are sufficiently available for practical applications.

Sea buckthorn leaves are rich in various nutrients and bioactive compounds, including flavonoids, phenolic acids, proanthocyanidins, fatty acids, triterpenes, vitamins, and phytosterols, and have been reported to help prevent obesity, dyslipidemia, and atherosclerosis, in addition to exhibiting antioxidant activity [4,16]. Currently, sea buckthorn leaves are mainly used to produce sea buckthorn leaf tea, the primary product available on the market, which has garnered increasing interest in recent years due to its notable health benefits [18,19,20]. It has been reported that sea buckthorn leaf Fu tea, prepared by fermenting sea buckthorn leaves with *A. cristatus*, exhibited significantly enhanced antioxidant activity and lipid-lowering activity [4], opening new avenues for high-value utilization of sea buckthorn leaves. However, the previous research only attempted to produce fermented sea buckthorn leaf tea through single-strain fermentation with *A. cristatus.* Although *A. cristatus* is the key microorganism required for FBT production, a microbial environment composed of both bacteria and fungi is important for the practical production of FBT [21,22]. Consequently, in a previous preliminary study, we successfully applied practical FBT processing techniques (with technical support from Yiyang Tea Processing Factory Co., Ltd. (Yiyang, Hunan Province, China)) to produce Fuzhuan brick sea buckthorn leaf tea. Our previous study indicated that this process effectively improved the flavor profile and volatile composition of sea buckthorn leaf tea [23]. The important roles and succession of microorganisms in FBT have been well-studied [24]. However, no related report is currently available on microbial succession during the fermentation of sea buckthorn leaf tea. Understanding the microbial succession is essential for advancing the development of novel plant-based beverages. Therefore, this study investigated the dynamics and diversity of bacterial and fungal community succession during the solid-state fermentation of sea buckthorn leaf tea using FBT processing techniques. By elucidating how this unique plant matrix drives specific community assembly, our findings broaden the understanding of food microbiology in untraditional substrates. Furthermore, identifying distinct temporal stages of microbial restructuring provides actionable targets for precise fermentation control. Tracking the compositional decline of putative opportunistic taxa establishes an ecological baseline to inform future safety assessments, while identifying persistently dominant fungi explores their potential as process biomarkers to facilitate future production standardization.

## 2. Materials and Methods

### 2.1. Materials and Reagents

Sea buckthorn leaf green tea was prepared from sea buckthorn leaves collected in Huangyuan County, Qinghai Province, China, according to the manufacturing procedures for green tea (pan-frying, rolling, and drying). The production of Fuzhuan brick sea buckthorn leaf tea was carried out according to the processing techniques of FBT with technical support from Hunan Yiyang Tea Processing Factory Co., Ltd., Yiyang, China. Briefly, the sea buckthorn leaf green tea was first conveyed via a conveyor belt to a high-temperature steam-spraying zone for treatment. Subsequently, the steamed tea was wrapped in cloth and subjected to 3 h of pile fermentation. After pile fermentation, an appropriate amount of water was added to the tea sample and thoroughly mixed to maintain its moisture content at approximately 18%. The moistened tea was then pressed into brick-shaped molds to produce tea bricks (weighing about 500 g per brick). After shaping, the tea samples were transferred to a fermentation chamber for 15 days of fermentation. The fermentation conditions were set as follows: during the initial 0–3 days, the temperature was 27 °C with 50% humidity; during the middle phase, the temperature was 28 °C with approximately 60% humidity; during the final 2 days, the temperature was 29 °C with approximately 60% humidity. Starting from day 0, tea samples were collected every three days until fermentation was completed (Table 1). These samples were stored at −80 °C for subsequent experiments. To ensure biological reproducibility and the robustness of the findings, four independent fermentation batches of Fuzhuan brick sea buckthorn leaf tea were conducted simultaneously under identical environmental conditions. These batches served as four distinct experimental units. At each designated time point (Table 1), one sample was collected from each independent batch, resulting in four biological replicates per sampling interval. To capture and preserve the inherent biological variability, microbial genomic DNA extraction, library construction, and Illumina NovaSeq 6000 (San Diego, CA, USA) sequencing were performed independently for each individual replicate.

MagBeads Soil Genomic DNA Extraction Kit was obtained from MP Biomedicals, LLC. (Santa Ana, CA, USA). All chemical reagents were of commercially available analytical grade.

### 2.2. DNA Extraction

Each Fuzhuan brick sea buckthorn leaf tea sample (10 g) was suspended in 200 mL of sterile physiological saline solution (0.85% NaCl) and shaken with 3 mm glass beads at 200 rpm for 30 min in a shaker (room temperature). After that, the suspension was filtered through two layers of sterile gauze, and the resulting filtrate was centrifuged at 4°C and 10,000 rpm for 15 min to obtain the microbial pellet. To minimize the co-precipitation of interfering substances (e.g., tea proteins and pigments) while mitigating potential microbial loss, the pellet was subjected to five sequential washes with sterile physiological saline (0.85% NaCl). This washing protocol was adapted from established methods for the isolation of microbes from complex plant matrices [25,26]. The washing conditions (volume, time, and speed) were kept consistent across all samples to prevent processing bias. Microbial genomic DNA for the analysis of bacterial and fungal diversity in Fuzhuan brick sea buckthorn leaf tea was extracted from the pellet using the MagBeads Soil Genomic DNA Extraction Kit in accordance with the manufacturer’s instructions.

### 2.3. PCR Amplification, Illumina NovaSeq 6000 Sequencing and Data Quality Control

The high-throughput sequencing of the extracted genomic DNA was conducted by Shanghai GeneCowin Biotechnology Co., Ltd. (Shanghai, China) on an Illumina NovaSeq 6000 platform. Briefly, the extracted genomic DNA was quantified using a Qubit 3.0 fluorometer (Thermo Scientific, Waltham, MA, USA), and its integrity was assessed by 1.5% agarose gel electrophoresis. The hypervariable V3-V4 region of bacterial 16S rDNA was amplified using the primers 341F (5′-CCTACGGGNGGCWGCAG-3′) and 805R (5′-GACTACHVGGGTATCTAATCC-3′). For fungi, the ITS region was amplified using the primers ITS1 (5′-CTTGGTCATTTAGAGGAAGTAA-3′) and ITS2 (5′-GCTGCGTTCTTCATCGATGC-3′). After amplification, PCR products were detected by agarose gel electrophoresis. Triplicate PCR products from the same sample were pooled, and the pooled samples were used as templates for library construction. High-fidelity PCR was performed with primers containing index sequences to introduce Illumina-compatible tag sequences at the ends of library. Amplified products were detected by gel electrophoresis and purified using the Agencourt AMPure XP Kit (Beckman Coulter, Brea, CA, USA). Library quality was assessed using a Qubit 3.0 fluorometer and an Agilent Bioanalyzer 2100. Libraries were sequenced on an Illumina NovaSeq 6000 sequencer to generate raw reads. Raw sequencing reads were processed using QIIME2 framework (version 2023.2). Firstly, the cutadapt plugin was employed to remove adapter and primer sequences (trim-paired--p-error-rate 0.15 p-trunc-len-f/r = 220, p-trim-left-f/r = 0). Subsequently, the raw data underwent denoising, assembly, and de-chimerization via the DADA2 plugin to generate high-quality ASV (Amplifier Sequence Variant) feature tables and representative sequences (denoise-paired MaxEE = 2, truncQ = 2, p-chimera-method = consensus, p-min-fold-parent-over-abundance = 1.0). Taxonomic annotation of ASVs was performed using the pre-trained classifier (classify-sklearn--p-confidence 0.8) against the SILVA database (version 138.2) and UNITE (version 10.0), with confidence thresholds of 0.8 for 16S rRNA genes and 0.6 for ITS region.

All taxonomic analyses of microbial community data in this study adhered to the latest nomenclature standards established by the International Committee on Systematics of Prokaryotes (ICSP). Accordingly, this paper uniformly employed the revised phylum-level nomenclature, including Pseudomonadota (corresponding to the former Proteobacteria) and Bacillota (corresponding to the former Firmicutes). Names of other higher taxonomic units mentioned herein were also standardized according to this system.

### 2.4. Statistical Analysis

Experimental data are presented as the mean ± standard deviation (SD) of four independent replicates per condition. For multiple comparisons, one-way analysis of variance (ANOVA) followed by Tukey’s test using SPSS 22 software (SPSS Inc., Chicago, IL, USA) was performed. For non-parametric data, the Kruskal–Wallis rank-sum test was applied. In both analyses, a *p* value < 0.05 was considered statistically significant. Differences in microbial community structures across fermentation stages were evaluated using permutational multivariate analysis of variance (PERMANOVA) based on Bray–Curtis distances with 999 permutations. This analysis was executed via the vegan package (version 2.7.2) in R software (version 4.5.1; Foundation for Statistical Computing, Vienna, Austria). Post-hoc pairwise comparisons were conducted using the pairwiseAdonis package. To control for the inflation of Type I errors, *p* value was adjusted using the false discovery rate (FDR, Benjamini–Hochberg) method, with statistical significance defined as an adjusted *p*-value < 0.05. Moreover, correlation analysis was conducted using Spearman’s rank correlation method (|ρ| ≥ 0.6 and adj.*p* ≤ 0.01).

## 3. Results and Discussion

### 3.1. Richness and Diversity of Bacteria and Fungi

The richness and diversity of microorganisms are central to the formation of unique flavor and quality of Fuzhuan brick sea buckthorn leaf tea. In this study, the bacterial and fungal communities in Fuzhuan brick sea buckthorn leaf tea were analyzed by Illumina NovaSeq 6000 sequencing. For bacteria, 2,284,108 sequences were generated from the 24 tea samples. After quality control processing, including filtration, denoising, merging, and non-chimerism, 1,870,134 high-quality sequences were obtained. For fungi, 1,672,076 raw sequences were generated, yielding 1,489,511 high-quality sequences after quality control.

**Figure 1:**
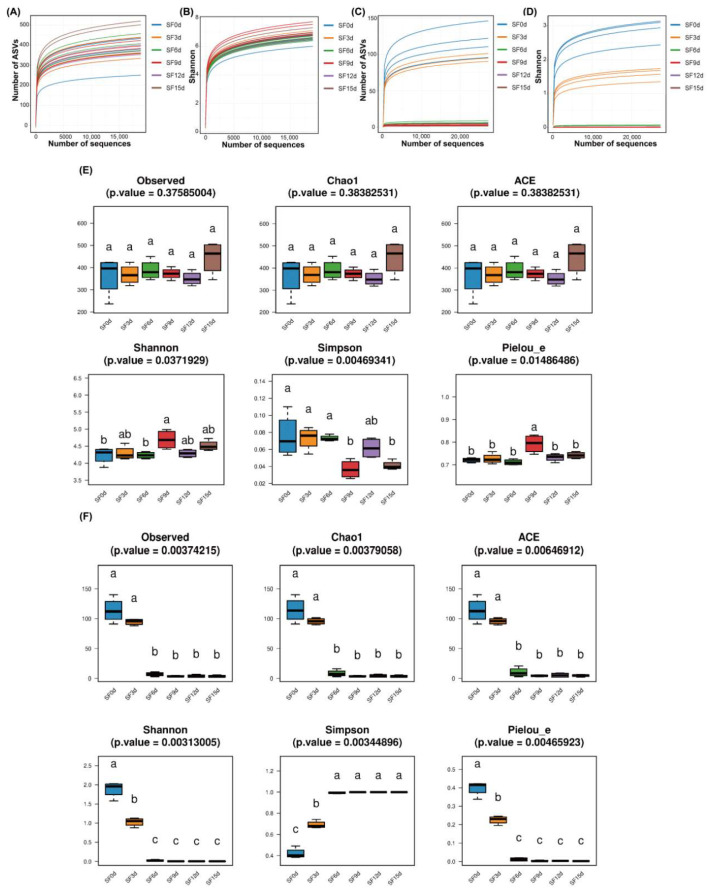
Impact of fermentation on microbial communities’ α diversity of Fuzhuan brick sea buckthorn leaf tea. Rarefaction curves of bacterial communities (**A**), Shannon curves of bacterial communities (**B**), Rarefaction curves of fungal communities (**C**), Shannon curves of fungal communities (**D**), Diversity indexes of bacteria (**E**) and fungi (**F**). (**C**,**D**), the rarefaction and Shannon curves of several samples (notably SF12d, and SF15d) exhibit extensive overlapping. This is due to the high similarity in fungal community diversity during the late stages of fermentation and does not hinder the scientific understanding of the successional trends. Intergroup diversity in diversity indices was analyzed using the Kruskal-Wallis rank-sum test, with *p* < 0.05 considered statistically significant. Multiple hypothesis testing correction was performed using the Bonferroni method. Different lowercase letters above the bars indicate significant differences in species diversity among groups (*p* < 0.05); groups sharing the same letter are not significantly different.

Rarefaction curves of the bacterial communities gradually approached saturation (Figure 1A), whereas the Shannon curves did not reach a plateau (Figure 1B). However, the coverage rate of each sample reached 99.96 ± 0.02% (Table S1), indicating that the sequencing depth was sufficient to capture most bacterial taxa. Similarly, the coverage of fungi reached 99.99 ± 0.01%, indicating sufficient sequencing depth (Table S2). As sequencing depth increased, neither the Rarefaction curves (Figure 1C) nor Shannon curves (Figure 1D) for samples of SF0d and SF3d fully reached a plateau. However, after day 6 of fermentation, the Rarefaction curves and Shannon curves for all tea samples stabilized. Although sequencing coverage was high, the fact that several bacterial Shannon curves and some early-stage fungal curves did not fully reach plateau suggested that a fraction of low-abundance diversity might have remained undetected. This limitation mainly might affect the estimation of the rare biosphere rather than the dominant community structure, which was consistently resolved across samples [27]. Therefore, we could not exclude the presence of additional rare taxa with potential ecological functions, but this was unlikely to substantially change the main conclusions regarding community succession and dominant microbial enrichment during fermentation [3,24,28].

The α-diversity of microbial community was assessed using the Observed, Chao1, ACE, Shannon, Simpson, and Pielou_e indices. α-Diversity refers to the richness (number of taxa) or evenness (relative abundance of these taxa) observed in the microbiome. The first three indices were used to calculate species richness, while the latter three were employed to represent evenness [29,30]. Intergroup diversity in diversity indices was analyzed using the Kruskal–Wallis rank-sum test, with *p* < 0.05 considered statistically significant. Multiple hypothesis testing correction (fdr) was performed using the Bonferroni method to assess significant difference in species diversity among groups. As shown in Table S1, the Chao1 values of bacterial communities ranged from 351.78 ± 32.40 to 446.13 ± 75.94, while the ACE values ranged from 351.47 ± 31.71 to 446.02 ± 75.68. The Shannon index ranged from 4.23 ± 0.25 to 4.69 ± 0.28, and the Simpson index ranged from 0.04 ± 0.01 to 0.08 ± 0.03. No significant differences were found in the Observed, Chao1, or ACE indices among the samples. No significant differences were found in Observed ASVs, Chao1, or ACE among the different stages of fermentation, indicating that fermentation did not substantially alter overall bacterial richness. In contrast, bacterial evenness showed stage-dependent variation. Significant differences in Shannon, Simpson, and Pielou_e indices were observed, with SF9d showing a more even community structure than the other stages (Figure 1E).

In contrast to the bacterial community, fungal α-diversity decreased markedly during fermentation. The number of fungal ASVs declined from 113 ± 21 (SF0d) to 94 ± 5 (SF3d) and ultimately to 6 ± 3 (SF6d). Compared with SF0d, the number of ASVs decreased significantly as fermentation progressed. The Chao1 values for fungal communities across all samples ranged from 3.50 ± 1.91 to 114.56 ± 20.70 (Table S2), while ACE values ranged from 4.43 ± 1.30 to 114.11 ± 20.51. The Shannon index ranged from 0.00 ± 0.00 to 1.88 ± 0.21 across all samples, while the Simpson index ranged from 0.42 ± 0.05 to 1.00 ± 0.00. Similarly, Chao1, ACE, Shannon, and Pielou_e values decreased sharply, whereas the Simpson index increased to nearly 1.0 (Figure 1F), indicating that during the initial fermentation stage (0–6 d), the richness and evenness of the fungal community in the Fuzhuan brick sea buckthorn leaf tea rapidly decreased. Subsequently, no significant difference in α-diversity of fungal community was observed. The sharp decline in fungal diversity during the early stage of fermentation likely reflects a technologically driven stabilization process. In Fuzhuan brick tea and related “golden flower” fermentation, the rapid dominance of specific fungi is generally regarded as a hallmark of proper process progression. This selective enrichment may restrict ecological niches available to spoilage or toxigenic fungi, thereby potentially enhancing fermentation consistency and influencing microbial safety profiles [1,31]. Furthermore, the prevailing *Eurotium*/*A. cristatus* acts as the primary functional driver in this system, mediating the synthesis of characteristic metabolites and health-promoting compounds [21,32]. Consequently, the simplified fungal community suggests a shift toward a highly specialized microbiome, which may hypothetically be adapted for the target characteristics of the fermented product.

Nevertheless, this taxonomic simplification raises legitimate concerns regarding a potential loss of biotransformation complexity, since the depletion of minor fungal taxa could restrict synergistic enzymatic networks [33]. Despite this, overall fermentation functionality is unlikely to be substantially impaired. The persistent dominance of *A. cristatus* suggests robust metabolic specialization, while the consistently high bacterial richness observed throughout fermentation likely compensates for the reduced fungal complexity. Previous studies have demonstrated that bacterial communities actively drive carbohydrate degradation, polyphenol conversion, and aroma development in Fuzhuan brick tea, particularly during the initial stages [6,28,34]. Thus, the early fungal stabilization is consistent with a shift toward a specialized microbiome, which may facilitate consistent fermentation under industrial conditions.

### 3.2. Microbial β-Diversity of Bacteria and Fungi

**Figure 2:**
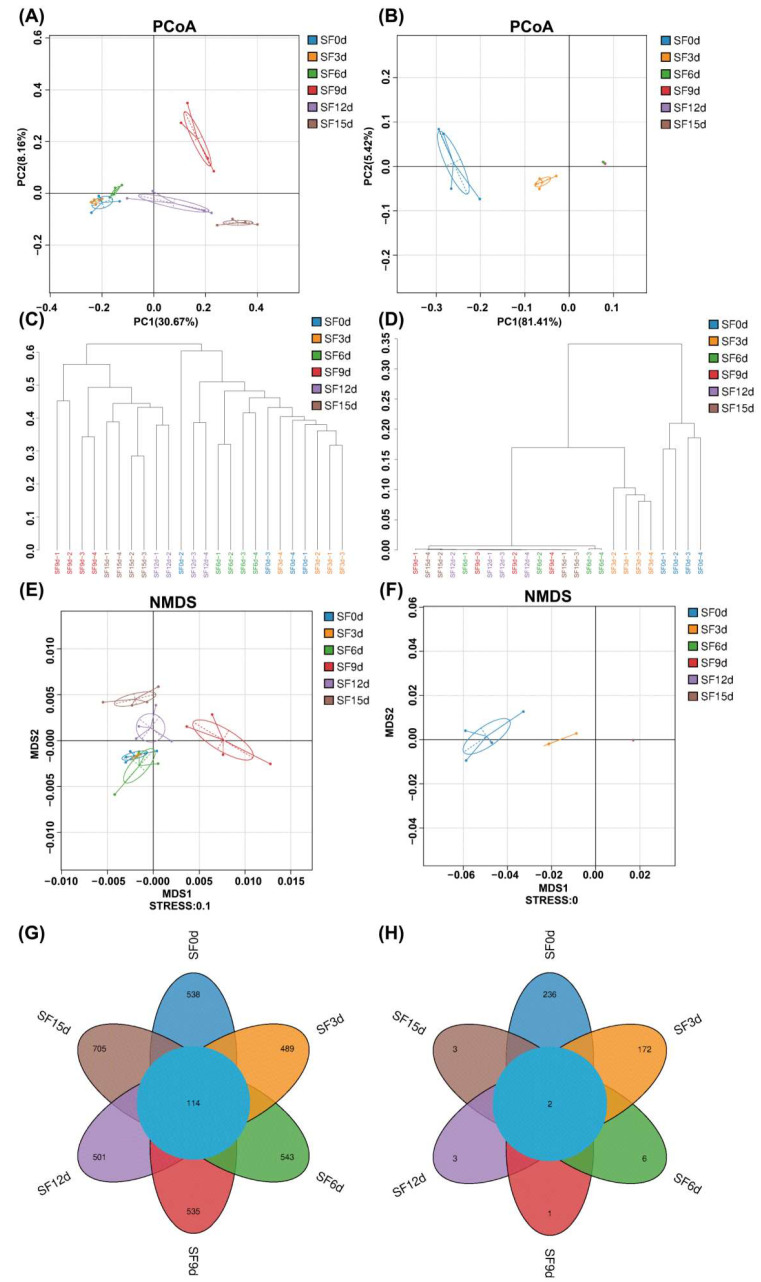
Analysis of microbial communities in Fuzhuan brick sea buckthorn leaf tea samples during fermentation. PCoA analysis of bacteria (**A**) and fungi (**B**), based on Bray–Curtis distances between samples, the horizontal axis represents one principal component, and the vertical axis represents another principal component. Percentages indicated the contribution of each principal component to the variation between samples; Clustering tree diagrams for bacterial communities (**C**) and fungal communities (**D**), branch length indicates species composition similarity and shorter branches indicate greater similarity between samples; NMDS analysis based on weighted unifrac of bacteria (**E**) and fungi (**F**): Ellipses (indicated by solid and dashed lines) are drawn to encircle the data points of each group, highlighting the spatial clustering and dispersion boundaries of the microbial communities at different fermentation stages. In panels (**B**,**F**), certain colors listed in the legend (e.g., SF9d, SF12d, and SF15d) may not be individually distinguishable because the data points overlap almost entirely. This overlapping is a direct result of the high structural convergence and low diversity of the fungal communities in the late fermentation stages; Venn diagram of bacterial (**G**) and fungi (**H**) communities.

To analyze microbial β-diversity during the fermentation of sea buckthorn leaf tea, principal coordinate analysis (PCoA) and non-metric multidimensional scaling (NMDS), and cluster tree analysis were employed to visualize the evolution of microbial communities. PCoA based on Bray–Curtis distances effectively visualized compositional differences in microbial community. It revealed distinct differences in bacterial communities among groups during the fermentation of sea buckthorn leaf tea. The first principal coordinate (PC1) and second principal coordinate (PC2) explained 30.67% and 8.16% of the variance, respectively (Figure 2A). The fungal communities across different groups showed a dispersed distribution in the PCoA (Figure 2B), with PC1 explaining 81.41% of the variance and PC2 explaining 5.42%.

Cluster tree analysis provided an overall description and comparison of the similarities and differences among samples throughout the fermentation process [35]. Similar results were observed in the cluster tree analysis. The six bacterial groups evolved separately during community succession (Figure 2C). The SF9d group showed distinct differences from the other groups, while the SF0d, SF3d, and SF6d groups clustered more closely together. During the fungal community evolution, SF0d and SF3d not only showed distinct differences from other groups, but also exhibited significant divergence from each other, whereas the remaining four groups were more closely related (Figure 2D).

NMDS based on weighted Unifrac distances further revealed the evolution of microbial communities during fermentation. NMDS results for bacterial communities indicated smaller ellipse areas for SF3d, SF6d, and SF15d samples, suggesting greater stability in community composition during the early and late fermentation stages (Figure 2E). The larger ellipses for SF9d and SF12d suggested more pronounced community fluctuations during the mid-fermentation phase. The isolated position of SF9d indicated that the ninth day of fermentation might represent a critical period for community restructuring. Additionally, bacterial community succession proceeded more slowly during the early fermentation stage (0–6 days) compared to the late fermentation stage (9–15 days), when more pronounced changes in community structure were observed. The NMDS analysis of fungal communities showed a progressive rightward shift from SF0d to SF15d, forming a distinct gradient distribution (Figure 2F). SF0d exhibited the greatest divergence from other time points, consistent with its unfermented initial state. SF3d began to separate significantly, potentially reflecting the replacement of dominant microbial communities during early fermentation (e.g., transition from environmental microbes to fermentation-specific microorganisms). The points for SF6d, SF9d, SF12d, and SF15d tended to cluster together, indicating that the fungal community structure of these samples had entered a relatively stable phase. The results from PCoA, NMDS, and cluster tree analysis were consistent, providing multi-faceted validation of the reliability of the findings. Post-hoc pairwise PERMANOVA further revealed the distinct successional rhythms between bacterial and fungal communities (Table S3). For bacterial communities, sequential shifts were statistically supported across multiple fermentation stages. Conversely, the fungal community exhibited a contrasting pattern. During the initial fermentation stages (from day 0 to day 6), the fungal community underwent drastic restructuring, evidenced by exceptionally high explanatory power (*R*^2^ ranging from 0.58 to 0.80). Although these comparisons did not consistently reach statistical significance after FDR correction (adjusted *p* = 0.055), likely owing to the limited sample size (n = 4) and corresponding permutation constraints [36], these large *R*^2^ values still indicated substantial community replacement [37]. Notably, in the mid-to-late stages (from day 6 to day 15), the inter-group variations dramatically decreased (*R*^2^ ≤ 0.32, adj.*p* ≥ 0.240), indicating that the fungal community, dominated by *Aspergillus*, had entered a highly stable equilibrium. All the results of PCoA, NMDS, and tree cluster analyses indicated differences in microbial composition among samples from various fermentation stages. The fungal community structure during the fermentation of sea buckthorn leaf tea showed marked temporal variation, progressing through three distinct phases: an initial adaptation phase, a rapid change phase, and a stabilization phase.

According to the Venn diagram, throughout the fermentation process (0, 3, 6, 9, 12, and 15 d), the bacterial community shared 114 ASVs, and the total number increased from 538 to 705 (Figure 2G). In contrast, the fungal community shared only 2 ASVs (ASV1 and ASV76) across all fermentation stages, and the total number decreased from 236 to 3 (Figure 2H). Interestingly, ASV1 was identified as *A. chevalieri*, indicating that this species persisted throughout fermentation. The persistent dominance of *A. chevalieri* throughout fermentation is noteworthy. Sea buckthorn leaves are rich in bioactive phenolics, mainly flavonol glycosides and ellagitannins; therefore, the sea buckthorn leaf matrix may exert a strong substrate-selection effect on fungal community assembly [19]. Compared with the tea leaves used for FBT processing, sea buckthorn leaves likely differ in indigenous microbiota, structural composition, and phytochemical profile, thereby favoring microorganisms that are better adapted to this specific solid-state fermentation environment [24,38]. In addition, the relatively high abundance of *A. chevalieri* in the unfermented sample (63.79% in SF0d) suggests a potential priority effect, whereby an initially abundant fungus may rapidly colonize the substrate, utilize available nutrients through extracellular enzymatic activity, and maintain ecological dominance during fermentation [33,39].

This pattern also appears to differ from the classic fungal succession reported for FBT produced from tea-based substrates [40]. Previous studies have shown that conventional FBT fermentation is characterized by a complex microbial community, including *Debaryomyces*, *Aspergillus*, and *Klebsiella* and is mainly driven by *Eurotium* (*Aspergillus*) *cristatum* [24]. In contrast, in the present study, *A. chevalieri* was already highly abundant before fermentation and remained dominant throughout the process, indicating that FBT-like processing applied to sea buckthorn leaves does not simply reproduce the canonical microbial trajectory observed in traditional tea fermentation. Rather, the substrate itself appears to substantially redirect microbial succession.

This substrate-dependent dominance may also reflect differences in the metabolic and ecological specialization of *A. chevalieri* relative to *E. cristatum*. Although direct comparative metabolomic studies of these two fungi remain limited, the available literature indicates that both possess active secondary metabolism, but with different documented emphases. *E. cristatum*, the canonical fungus in conventional FBT, has been reported to produce anthraquinones and related metabolites, including citreorosein, emodin, physcion, echinulin, flavoglaucin, auroglaucin, and aspergin, which are closely associated with tea fermentation and tea-specific metabolite transformation [41,42]. By contrast, *A. chevalieri* has been reported to produce alkylated salicylaldehydes and prenylated indole alkaloids, and the studied strain is isolated from a host-associated endolichenic microenvironment, suggesting, at least for some strains, an ability to persist in chemically complex biological substrates [43]. These observations suggest that the difference between the two fungi may lie less in overall metabolic capacity than in functional emphasis and ecological adaptation. Given that sea buckthorn leaves are enriched in phenolics and other bioactive constituents [19], these substrates may impose stronger chemical selectivity than tea leaves and thus provide a more favorable niche for *A. chevalieri* than for the tea-associated *E. cristatum*. Therefore, the predominance of *A. chevalieri* in the present system is more likely to reflect substrate-driven ecological specialization than a simple taxonomic substitution within an otherwise unchanged FBT fermentation trajectory.

The high initial abundance of *A. chevalieri* is consistent with, but does not conclusively prove, an endophytic origin. Endophytes are microorganisms that colonize internal plant tissues for at least part of their life cycle without causing apparent disease symptoms [44]. Therefore, although the presence of *A. chevalieri* before fermentation supports the possibility that it may originate from the sea buckthorn leaf itself, amplicon sequencing alone cannot distinguish true endophytes from epiphytic fungi or microorganisms introduced during harvesting and handling. Nevertheless, *Aspergillus* species have frequently been reported as endophytic fungi in plants, and bioactive metabolites produced by *A. chevalieri* have been shown to possess antibacterial and antioxidant activities [43,45], which may partly explain its ecological competitiveness in this fermentation system. Fermentation of sea buckthorn leaf tea induced specific changes in the microbial community, and fermentation time significantly affected the structure and abundance of microorganisms present. This may substantially influence the quality of fermented sea buckthorn leaf tea.

### 3.3. Dynamic Changes in Bacterial and Fungal Community Composition

**Figure 3:**
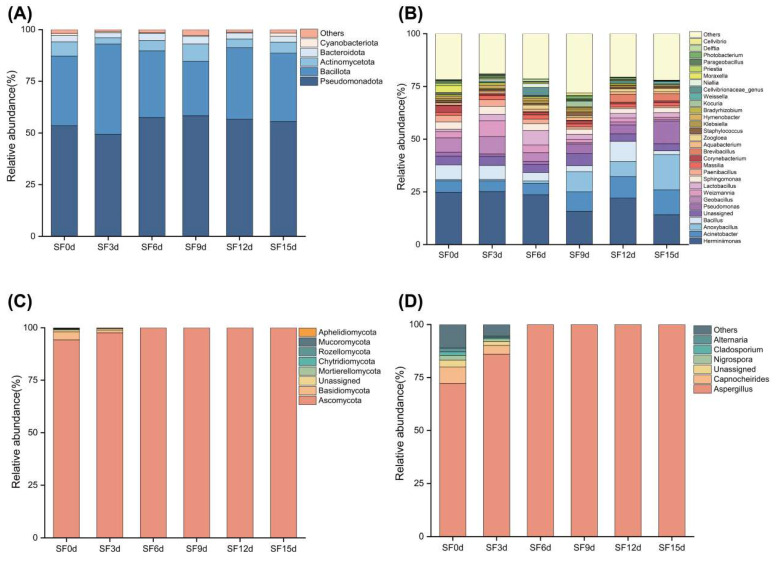
Microbial community composition. Relative abundance at the phylum, genus level of bacteria (**A**,**B**) and fungi (**C**,**D**).

The distinctive flavor of fermented tea is primarily produced through microbial action during the manufacturing process [33]. At the phylum level of bacterial communities, Pseudomonadota, Bacillota, and Actinomycetota were the dominant phyla (Figure 3A). *Herminiimonas*, *Acinetobacter*, *Anoxybacillus*, *Bacillus*, *Pseudomonas*, *and Geobacillus* were the dominant genera among bacterial community. The genus *Acinetobacter* showed an increasing trend, with relative abundance rising from 5.52% to 11.85% as fermentation progressed. The genus *Anoxybacillus* exhibited fluctuating increases, with relative abundance climbing from 4.97% to 16.73%. The *Pseudomonas* genus showed a marked increase, with relative abundance rising from 1.85% to 10.67%. The increased abundance of *Pseudomonas* may also be relevant to flavor development during fermentation. Members of the genus *Pseudomonas* are known for their metabolic versatility and have been associated with the formation or transformation of volatile compounds, including aldehydes, alcohols, ketones, and esters, through pathways related to amino acid catabolism, lipid degradation, and oxidative metabolism in plant-associated microbial systems [46,47]. Similarly, previous studies on FBT have identified *Pseudomonas* as a predominant bacterial genus that demonstrates an overall increasing trend in relative abundance throughout the fermentation process [34], and its presence has been linked to the generation of odor-active compounds [28]. The relative enrichment of *Pseudomonas* observed in our system aligns well with the previous study, suggesting a potential role in volatile development. However, its specific contribution to the aroma profile of Fuzhuan brick sea buckthorn leaf tea remains a hypothesis that requires future validation via direct metabolomic coupling. Conversely, the genera *Herminiimonas* and *Geobacillus* exhibited overall decreasing trends, with relative abundances falling from 24.79% and 6.83% to 14.12% and 0.87%, respectively (Figure 3B). The dominant bacterial phyla in FBT made from tea leaves are Pseudomonadota, Bacillota, and Actinomycetota [28], and the characteristic bacterial genera in FBT are *Bacillus*, *Lactococcus*, *Klebsiella*, and *Pseudomonas* [1]. The composition and structure of bacterial communities significantly influence tea quality [28]. Compared with FBT, the differences in the dominant bacterial genera in Fuzhuan brick sea buckthorn leaf tea might be due to differences in raw materials, as FBT is made from the leaves of *Camellia sinensis*, whereas Fuzhuan brick sea buckthorn leaf tea is made from the leaves of *Hippophae rhamnoides*. Furthermore, the bacterial community compositions of fermented teas produced by different processing techniques also differ. For instance, *Staphylococcus*, *Bacillus*, *Klebsiella*, and *Bacillus subtilis* are the predominant bacterial genera during the aging process of Pu-erh tea and Liubao tea [48].

Ascomycota and Basidiomycota were the two phyla within the fungal community, with Ascomycota being the predominant fungal group across all samples. Ascomycota constituted more than 94.2% of the SF0d sample and continued to increase throughout the fermentation process, whereas Basidiomycota accounted for only 3.8% and continued to decrease as fermentation progressed (Figure 3C). *Aspergillus* was the predominant genus within the fungal community. During the initial fermentation stage, *Capnocheirides*, *Nigrospora*, *Cladosporium*, and *Alternaria* were detected. Although *Aspergillus* exhibited a relative abundance of 72.2% during the initial fermentation stage and continued to increase as fermentation progressed, the relative abundances of the other fungal genera decreased throughout the process. Throughout the entire fermentation process, *Aspergillus* consistently maintained a high relative abundance and retained its dominant position (Figure 3D).

The characteristic fungal genera of FBT mainly include *Aspergillus*, *Debaryomyces*, and *Cyberlindnera*. Among them, only *Aspergillus* maintained dominance and relative stability throughout the whole manufacturing process of Fuzhuan brick sea buckthorn leaf tea, which is consistent with the report [1]. The predominant fungus in FBT is commonly recognized as *E. cristatum*. Notably, species of *Eurotium* represent the teleomorphic (sexual) state of *Aspergillus*, whereas the asexual spore-producing structures are regarded as the distinguishing morphological and microscopic features of the genus *Aspergillus* [49]. Accordingly, the sexual and asexual morphs correspond to two different taxonomic descriptions but belong to the same fungal taxon. During fermentation, the dominant fungi in FBT display dual reproductive modes: the sexual state produces ascospores and corresponds to *E. cristatum*, whereas the asexual state corresponds to the conidial stages of *A. chevalieri*, *A. cristatus*, *A. cibarius*, and *A. amstelodami* [40].

### 3.4. Difference in the Microbial Community Composition in the Fermentation Process and Correlation of Differential Microorganisms

**Figure 4:**
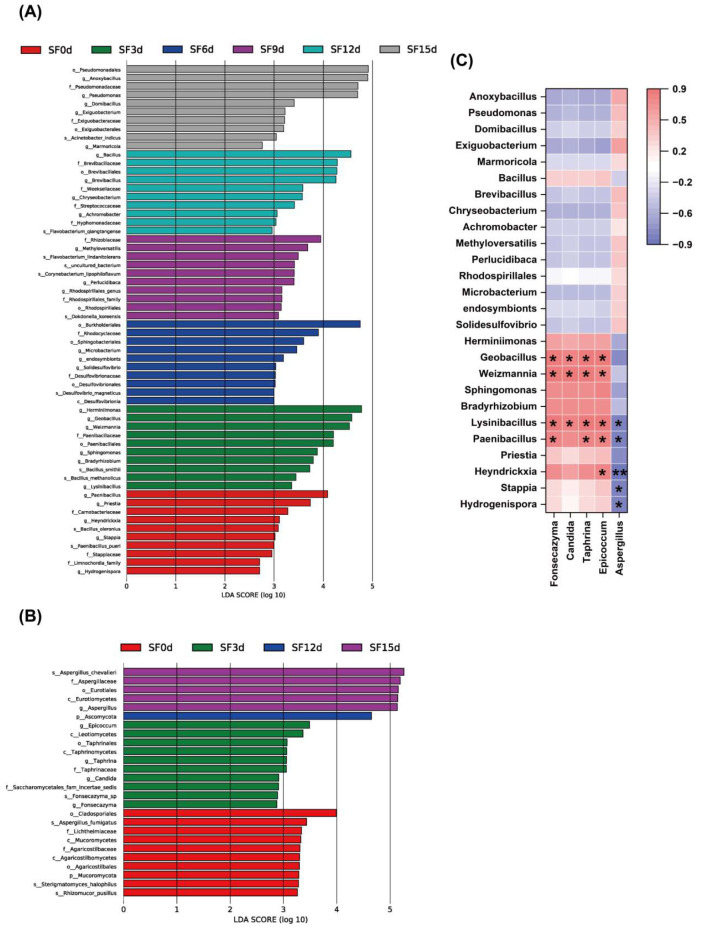
Significance of microbial differences between groups during fermentation. Characteristic bacteria (**A**) and fungi (**B**) by LEfSe analysis, and correlation between fungal community and bacterial community (**C**). The fill color indicates the direction of a correlation, red for positive and blue for negative. Correlation with *p* < 0.05 or 0.01 was displayed with * or **.

To elucidate the significant changes in microbial succession during fermentation, linear discriminant analysis effect size (LEfSe) analysis [50] was conducted, and the nonparametric Kruskal–Wallis rank-sum test was used to screen for species showing significant differences in relative abundance among groups. The key differentially abundant bacteria in SF0d were *Bacillus oleronius* and *Paenibacillus pueri*. It has been reported that *B. oleronius* is an inflammation-promoting species capable of inducing skin inflammation via the gut-skin axis [51,52]. The key differentially abundant bacteria in SF3d were *B. methanolicus* and *B. smithii*. *B. smithii* is a thermophilic spore-forming bacterium isolated from fermented products such as baijiu (Chinese liquor) and high-temperature compost. Previous studies have characterized *B. smithii* as a functional strain exhibiting gastric acid and heat tolerance, as well as resistance to certain pathogenic bacteria [53]. Although its enrichment during this phase suggests that it may contribute to the fermentation environment, its specific functional role and application potential in this tea require further empirical validation. The key differentially abundant bacterium in SF6d was *Desulfovibrio magneticus*, whereas the key differentially abundant bacteria in SF9d included *Dokdonella koreensis*, *Corynebacterium lipophiloflavum*, and *Flavobacterium lindanitolerans*. The key differentially abundant bacterium in SF12d was *F. qiangtangense*, whereas the key differentially abundant bacterium in SF15d was *Acinetobacter indicus* (Figure 4A).

Fungi constitute the most important microbial community in FBT. Differentially enriched fungi in SF0d included Mucoromycota, Cladosporiales, Agaricostilbales, and Lichtheimiaceae. The SF3d group showed enrichment of *Epicoccum*, *Taphrina*, and *Candida* (a genus containing known opportunistic fungi) [54]. The presence of *Candida* in the SF3d sample is consistent with the previous finding that *Aspergillus*, *Cyberlindnera*, and *Candida* are commonly present during the earliest stage of flowering fermentation in FBT [1]. However, throughout the production process of FBT, only *Aspergillus* persists [39]. The SF12d sample exhibited enrichment of Ascomycota, whereas the SF15d sample showed enrichment of Eurotiales and *A. chevalieri* (Figure 4B). In a previous report, *A. chevalieri* found in FBT was also detected in the plant *Camellia sinensis*, which was used for the production of Liupao tea and FBT [55].

Additionally, based on LEfSe analysis results, a genus-level correlation analysis was performed for microorganisms showing significant differences in abundance among the samples (Figure 4C). Data from all samples were used to analyze Spearman correlations between fungal and bacterial communities [26]. Correlation analysis of fungi and bacteria was conducted to explore statistical associations compatible with possible promoting, antagonistic, or competitive roles during fermentation [56]. At the genus level, the dominant fungus *Aspergillus* exhibited significant negative correlations with four bacterial genera, *Lysinibacillus*, *Paenibacillus*, *Stappia*, and *Hydrogenispora*, and a highly significant negative correlation with *Heyndrickxia*. Several other fungi exhibited significant positive correlations with certain bacteria: *Fonsecazyma* showed significant positive correlation with 4 bacterial taxa, *Candida* with 3, *Taphrina* with 4, and *Epicoccum* with 5. The growth of *Pseudomonas* and the dominant fungus *Aspergillus* showed a positive correlation during the fermentation.

Overall, LEfSe analysis revealed a progressive decline in the relative abundance of putative opportunistic taxa during fermentation. Specifically, the pro-inflammatory bacterium *B. oleronius* and the opportunistic fungus *Candida* were enriched only in the early stages (SF0d and SF3d). These taxa were not identified as characteristic features in the later stages. Their reduction is likely driven by three main mechanisms. First, the accumulation of organic acids may lead to environmental acidification [57]. Second, dominant microbes such as *Aspergillus* exert competitive exclusion [41]. Third, phenolic compounds released from sea buckthorn leaves exhibit inherent antimicrobial activity [58]. The present results suggested that, as fermentation progressed, the relative abundance of taxa often associated with tea fermentation increased, while the relative abundance of sequences assigned to taxa containing potential opportunistic strains (such as *Candida*) gradually decreased. While these compositional dynamics suggest that the fermentation environment may selectively restrict certain undesirable microbes, it must be stressed that such ecological shifts do not constitute direct evidence of final product safety. Rigorous absolute quantification and direct toxicological evaluations remain strictly necessary to confirm the safety profile of Fuzhuan brick sea buchthorn leaf tea. These dynamics suggest a potential pathway for influencing the quality of fermented products through microbial community regulation and provide further insights into the microbial ecosystem.

The relationships among core functional microorganisms involved in the natural fermentation of FBT remain poorly understood. Therefore, this study attempted to analyze statistical correlations among microorganisms showing significant intergroup differences. The negative correlation between *Aspergillus* and certain bacteria might stem from its ecological competitive advantage and the production of antimicrobial metabolites. The extracellular enzyme systems secreted by *Aspergillus* (e.g., cellulase and protease) might enable the efficient degradation of complex polymers in tea leaves, likely conferring a competitive advantage in the utilization of shared carbon and nitrogen sources and thereby inhibiting the growth of certain bacteria [24]. Antimicrobial secondary metabolites produced by *Aspergillus* could also directly inhibit the proliferation of susceptible bacteria [41,59]. Conversely, positive correlations between specific fungi and bacteria may reflect associations compatible with possible metabolic complementarity and cross-feeding among microorganisms. Such positive associations suggest hypotheses regarding potential functional division during complex substrate degradation. Bacterial pre-degradation might provide fungi with more readily accessible precursors, and vice versa, potentially forming reciprocal metabolic cross-feeding. In addition, positive correlations between specific bacteria and fungi might indicate micro-symbiotic relationships. Microorganisms with distinct metabolic networks may transform nutrients in tea leaf substrates to supply energy for detoxification processes, thereby improving the local microenvironment and hypothetically fostering localized mutualistic states [31,60].

**Figure 5:**
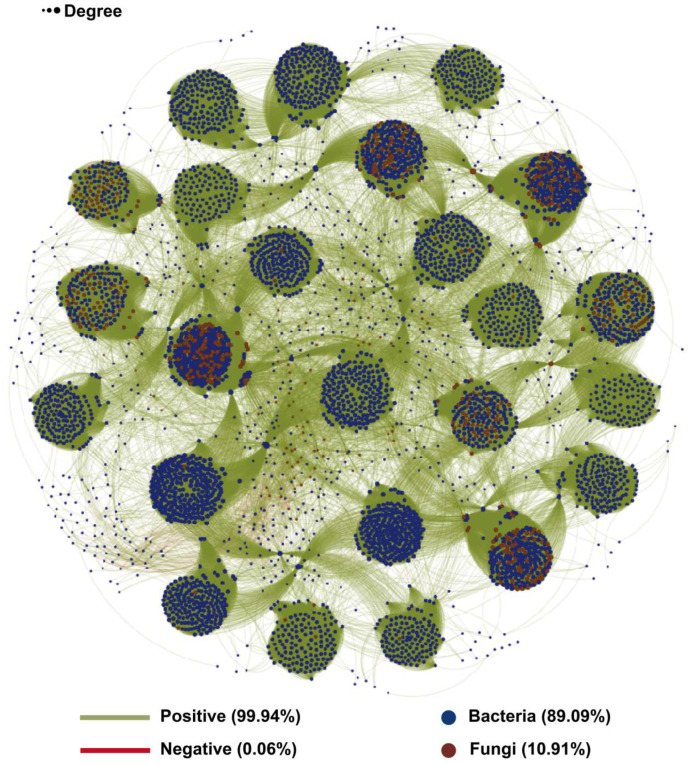
The bacteria and fungi association networks in the fermentation of Fuzhuan brick sea buckthorn leaf tea.

Correlations between microbial ASVs were calculated using Spearman’s rank correlation analysis. Only robust correlations (|ρ| ≥ 0.6 and adj.*p* ≤ 0.01) were retained for constructing co-occurrence networks between bacteria and fungi (Figure 5). This bacterial-fungal network comprised 4784 nodes and 371,583 edges, including 4262 bacterial nodes and 522 fungal nodes, indicating a substantially higher representation of bacteria than fungi. Among all edges, 66,833 edges were cross-kingdom connections, representing 17.99% of the total. Notably, 99.4% of these retained edges were positive. This pattern suggests that the fermentation system is dominated by co-occurrence relationships rather than mutually exclusive associations. However, it must be emphasized that these co-occurrence networks are strictly correlative and inferential. They do not necessarily indicate direct metabolic or ecological interactions, as positive correlations may also arise from shared niche preferences or similar responses to environmental changes. Nevertheless, the overwhelming predominance of positive associations is consistent with the possibility of metabolic cross-feeding within this system [61,62]. In plant-based solid-state fermentation, dominant fungi may decompose complex substrates, such as structural polysaccharides, proteins, and bound phenolic compounds, via extracellular enzymes [25,41]. This process releases low-molecular-weight metabolites that can be utilized by co-occurring bacteria and other fungi. In turn, these microorganisms may further transform such intermediates into secondary metabolites, vitamins, or organic acids, thereby forming a metabolically complementary network that supports community persistence and succession [25,26,63]. The results indicated that in the fermentation system of Fuzhuan brick sea buckthorn leaf tea, bacteria and fungi primarily exhibited associations compatible with possible cooperation rather than mutual exclusion. This extensive pattern of co-occurrence might contribute to maintaining the structural and functional stability of microbial communities within the system. To identify topologically important taxa, the top 10 nodes with the highest degree centrality were selected as hub nodes. The key fungal hubs were identified as Taphrina (ASV444) and *Selenophoma mahoniae* (ASV333), both belonging to the phylum Ascomycota. The key bacterial hubs spanned several phyla, including Actinomycetota (ASV858), Pseudomonadota (ASV2601, ASV1988, ASV964, ASV4321, ASV2048), and Bacillota (ASV698, ASV1679). Their central positions in the network suggest that these taxa may act as putative keystone members involved in maintaining network connectivity and mediating potential interspecies interactions [64,65].

### 3.5. Functional Analysis Using PICRUSt2

**Figure 6:**
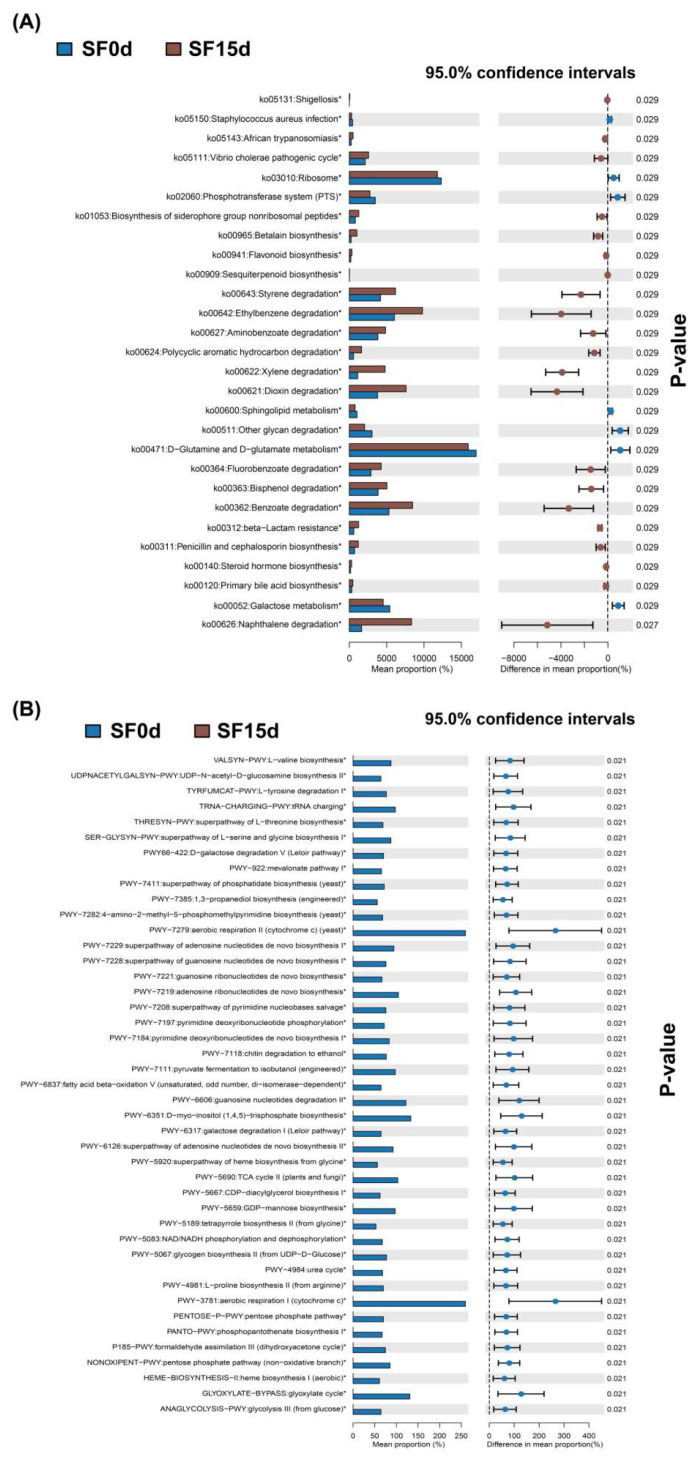
Using PICRUSt2 to predict microbial functions during the fermentation of bacteria (**A**) and fungi (**B**). Asterisks (*) indicate statistically significant differences in the relative abundance of metabolic pathways between the initial (SF0d) and final (SF15d) stages of fermentation (*p* < 0.05, Wilcoxon signed-rank test).

To predict microbial community functions and determine the relative abundances of functional genes, PICRUSt (Phylogenetic Reconstruction of Unobserved States for Community Studies) was employed. For bacterial communities, functional profiling was based on the KEGG (Kyoto Encyclopedia of Genes and Genomes) database using 16S rRNA gene sequences. Due to the limited availability of fungal genomic data, predicting fungal KEGG pathways remains challenging. Therefore, fungal metabolic pathways were inferred using the MetaCyc (Metabolic Pathways From all Domains of Life) database based on ITS sequences. Based on the PICRUSt2 prediction, the relative abundances of KEGG pathways in bacterial communities and MetaCyc pathways in fungal communities were calculated across the samples. A Wilcoxon signed-rank test was used to compare metabolic pathways in bacterial (Figure 6A) and fungal (Figure 6B) communities between the beginning and the end of fermentation, which identified the primary metabolic pathways associated with the fermentation process. It is important to clarify that this approach is inherently inferential and correlative, as it reconstructs functional profiles based on 16S/ITS marker-gene sequences rather than direct metabolic or transcriptomic measurements. KEGG pathways were categorized into three levels. At the primary level, the metabolism pathway was the most prominent, followed by genetic information processing. At the secondary level, xenobiotics biodegradation and metabolism was the primary pathway, supplemented by translation and carbohydrate metabolism pathways. At the tertiary level, D-glutamine and D-glutamate metabolism held the highest proportion, alongside pathways associated with the ribosome and the degradation of various aromatic compounds (e.g., styrene, ethylbenzene, aminobenzoate, xylene, dioxin, fluorobenzoate, bisphenol, benzoate, and naphthalene). In the fungal community, the most abundant MetaCyc pathways were PWY-7279 (aerobic respiration II [cytochrome c, yeast]) and PWY-3781 (aerobic respiration I [cytochrome c]). Other relatively important metabolic pathways included adenosine ribonucleotides de novo biosynthesis (PWY-7219), guanosine nucleotides degradation II (PWY-6606), D-myo-inositol (1,4,5)-trisphosphate biosynthesis (PWY-6351), and glyoxylate cycle (GLYOXYLATE-BYPASS).

Notably, functional prediction revealed a significant enrichment of several KEGG pathways categorized under xenobiotics biodegradation and metabolism, including benzoate and polycyclic aromatic hydrocarbon degradation. These annotations should not be interpreted as direct evidence for the presence of corresponding xenobiotic pollutants in sea buckthorn leaves, because PICRUSt2 infers functional potential from marker-gene profiles and KEGG pathway labels are defined according to representative model substrates rather than sample-specific chemical measurements [66,67]. Rather, they likely reflect the microbial capacity to metabolize structurally related plant-derived aromatic compounds, because many enzymes involved in xenobiotic degradation also participate in the catabolism of natural phenolics and lignin-derived metabolites [68,69].

We hypothesize that the most probable substrates for these pathways are aromatic compounds naturally abundant in sea buckthorn leaves. Sea buckthorn leaves are rich in isorhamnetin-, quercetin-, and kaempferol-based flavonol glycosides, as well as phenolic acids (gallic acid, protocatechuic acid, caffeic acid, ferulic acid, and p-coumaric acid) and condensed tannins [38,70], all of which provide aromatic substrates amenable to microbial ring oxygenation, cleavage, and side-chain transformation. Furthermore, fermentation-associated disruption of leaves and branches’ cell walls may release lignin-derived aromatic intermediates that can enter central bacterial aromatic catabolic routes [68]. Because monooxygenases, dioxygenases, and ring-cleavage enzymes involved in xenobiotic aromatic degradation commonly act on structurally analogous natural phenolics, the enrichment of these KEGG-annotated functions likely reflects enhanced microbial adaptation to the phenolic matrix of sea buckthorn leaves during fermentation rather than the degradation of exogenous contaminants. The enrichment of benzoate degradation is particularly noteworthy, as benzoate and protocatechuate represent central metabolic nodes shared across the microbial breakdown of diverse aromatic compounds, both natural and xenobiotic in origin [69]. Collectively, the increase in these predicted functions suggests an enhanced microbial potential for degrading and transforming the phenolic matrix of sea buckthorn leaves during fermentation. Crucially, this functional hypothesis aligns with recent empirical evidence regarding the specific metabolic capacities of *A. chevalieri* [71], the persistent and dominant fungus identified in our system. A recent study demonstrates that *A. chevalieri* possesses robust flavonoid-degrading and amino acid-synthesizing capacities during primary dark tea fermentation, effectively transforming complex phenolic compounds into metabolites associated with improved sensory quality [71]. Therefore, the sustained dominance of *A. chevalieri* suggests its potential role in the polyphenol-rich substrate transformation, which may contribute to quality modifications in fermented sea buckthorn leaf tea, a hypothesis that warrants further investigation via metabolomic analysis.

## 4. Conclusions

In conclusion, this study demonstrated that Fuzhuan brick sea buckthorn leaf tea could be produced as an FBT-like product through mixed solid-state fermentation following the manufacturing approach of FBT. During fermentation, the relative abundances of the bacterial genera *Acinetobacter*, *Anoxybacillus*, and *Pseudomonas* increased. As fermentation progressed, *Aspergillus* consistently retained its dominant position, and *A. chevalieri* persisted throughout the fermentation process. Microbial β-diversity analysis further indicated that bacterial community structure underwent greater change during the later fermentation stage (days 9–15), whereas the fungal community structure exhibited more pronounced change during the initial stage (days 0–3). In addition, although the dominant fungus *Aspergillus* exhibited significant negative correlations with five bacterial genera (*Lysinibacillus*, *Paenibacillus*, *Stappia*, *Hydrogenispora*, and *Heyndrickxia*), co-occurrence network analysis suggested an overall tendency toward coexistence rather than mutual exclusion between the bacterial and fungal communities. From an application perspective, these microbial dynamics provide a theoretical framework for optimizing Fuzhuan brick sea buckthorn leaf tea fermentation across four key domains. For fermentation control, the early fungal transition (days 0–3) and late bacterial restructuring (days 9–15) serve as critical windows for integrating targeted metabolomics with sensory profiling. In terms of safety assessment, the decline of putative opportunistic taxa (e.g., *Candida*) offers a positive ecological indicator, though direct toxicological validation remains necessary. Finally, the stable persistence of *A. chevalieri* indicates its potential as a process biomarker for future production standardization. However, several limitations of this study should be acknowledged. First, the microbial analysis relied primarily on amplicon sequencing and therefore characterized community composition rather than directly demonstrating the physiological activity or functional roles of individual taxa. Because no metabolomic or transcriptomic data were generated, the functional roles proposed for the identified ASVs remain hypothetical. Second, metabolites, bioactive compounds, and volatile aroma compounds were not quantitatively profiled, and no sensory evaluation was conducted; thus, the links between microbial succession and product quality remained preliminary. Third, the safety of the identified taxon *A. chevalieri* was not confirmed through strain isolation, culture-based characterization, or toxicological assessment. In addition, both the functional profiles inferred by PICRUSt2 and the associations derived from co-occurrence network analysis should be interpreted cautiously, as they are predictive and correlative rather than demonstrative of actual biological activities. To conclusively determine the functional roles of key taxa, particularly *A. chevalieri*, further research must prioritize strain isolation, controlled inoculation, and rigorous biosafety profiling. Subsequently, coupling targeted metabolomics with sensory validation will be essential to establish standardized quality control metrics for fermented sea buckthorn leaf tea.

## Figures and Tables

**Table 1:** Sample information of Fuzhuan brick sea buckthorn leaf tea.

Sample Name	Corresponding Sample Information
SF0d	The raw Fuzhuan brick sea buckthorn leaf tea
SF3d	The Fuzhuan brick sea buckthorn leaf tea fermented for 3 days
SF6d	The Fuzhuan brick sea buckthorn leaf tea fermented for 6 days
SF9d	The Fuzhuan brick sea buckthorn leaf tea fermented for 9 days
SF12d	The Fuzhuan brick sea buckthorn leaf tea fermented for 12 days
SF15d	The Fuzhuan brick sea buckthorn leaf tea fermented for 15 days

## Data Availability

The original contributions presented in this study are included in the article/Supplementary Materials. Further inquiries can be directed to the corresponding author.

## Supplementary Materials

The following supporting information can be downloaded at https://www.mdpi.com/article/10.3390/foods15101727/s1, Table S1. Statistical comparison of bacterial α-diversity indices and sequencing coverage during fermentation of Fuzhuan brick sea buckthorn leaf tea. Table S2. Statistical comparison of fungal α-diversity indices and sequencing coverage during fermentation of Fuzhuan brick sea buckthorn leaf tea. Table S3. Pairwise PERMANOVA results based on Bray–Curtis distance.
