# Supplementary material for: Dynamics and Diversity of Microbial Community Succession During the Solid-State Fermentation Process of Fuzhuan Brick Sea Buckthorn Leaf Tea
Source: Foods. 2026 May 14;15(10):1727. doi: 10.3390/foods15101727 (PMC13206053; doi:10.3390/foods15101727)
Supplement: Supplementary file 1 [file foods-15-01727-s001.zip › foods-4237961-supplementary.pdf]

Dynamics and diversity of microbial community succession during the solid-state fermentation process of Fuzhuan brick sea buckthorn leaf tea

Yulu Wang[+], Jialu Ao[+], Qiankun Guo, Zhiyong Xie, Xia Fan, Yi Sun, Zhipeng Wang, Jinghong Wei, Xiaoxiong Zeng [*]

College of Food Science and Technology, Nanjing Agricultural University, Nanjing, 210095, Jiangsu, China

[+] The authors contributed equally to this work. [*] To whom correspondence should be addressed. Tel & Fax: + 86 25 84396791, E-mail: zengxx@njau.edu.cn (X. Zeng)

Table S1. Statistical comparison of bacterial α-diversity indices and sequencing coverage during fermentation of Fuzhuan brick sea buckthorn leaf tea.

Samples	Chao1	ACE	Shannon	Simpson	Coverage
SF0d	364.80 ± 87.70	364.56 ±87.75	4.23 ±0.25^{b}	0.08 ±0.03^{a}	0.9997 ± 0.0001
SF3d	370.34 ±45.35	369.79 ± 45.03	4.29 ± 0.20^{ab}	0.07 ± 0.01^{a}	0.9997 ± 0.0001
SF6d	390.52 ± 45.93	390.07 ± 45.67	4.24 ± 0.10^{b}	0.07 ± 0.00^{a}	0.9997 ± 0.0001
SF9d	373.64 ± 25.19	373.43 ± 25.38	4.69 ± 0.28^{a}	0.04 ± 0.01^{b}	0.9999 ± 0.0001
SF12d	351.78 ± 32.40	351.47 ± 31.71	4.29 ± 0.11^{ab}	0.06 ± 0.01^{ab}	0.9999 ± 0.0002
SF15d	446.13 ± 75.94	446.02 ± 75.68	4.52 ± 0.15^{ab}	0.04 ± 0.01^{b}	0.9996 ± 0.0002

Values are presented as mean ± standard deviation (n = 4). Intergroup statistics were analyzed using the Kruskal-Wallis rank sum test, with $p < 0.05$ as the significance threshold. No significant differences were detected for Chao1, ACE, and Coverage among the fermentation stages.

Table S2. Statistical comparison of fungal α-diversity indices and sequencing coverage during fermentation of Fuzhuan brick sea buckthorn leaf tea.

Samples	Chao1	ACE	Shannon	Simpson	Coverage
SF0d	114.56 ± 20.70[a]	114.11 ± 20.51[a]	1.88 ± 0.21[a]	0.42 ± 0.05[c]	1.0000 ± 0.0001
SF3d	95.54 ± 5.28[a]	95.61 ± 5.39[a]	1.03 ± 0.11[b]	0.69 ± 0.04[b]	0.9999 ± 0.0001
SF6d	8.44 ± 5.56[b]	10.19 ± 7.78[b]	0.02 ± 0.02[c]	0.99 ± 0.01[a]	0.9999 ± 0.0001
SF9d	3.75 ± 0.96[b]	4.43 ± 1.30[b]	0.00 ± 0.01[c]	1.00 ± 0.00[a]	1.0000 ± 0.0000
SF12d	4.08 ± 2.59[b]	5.22 ± 3.74[b]	0.00 ± 0.00[c]	1.00 ± 0.00[a]	1.0000 ± 0.0000
SF15d	3.50 ± 1.91[b]	4.50 ± 2.29[b]	0.00 ± 0.00[c]	1.00 ± 0.00[a]	1.0000 ± 0.0000

Values are presented as mean ± standard deviation (n = 4). Intergroup statistics were analyzed using the Kruskal-Wallis rank sum test, with $p < 0.05$ as the significance threshold. No significant differences were detected for Coverage among the fermentation stages.

Table S3. Pairwise PERMANOVA results based on Bray-Curtis distance.

Comparison	df	Bacterial community			Fungal community		
		F	R^2	**adj. *p***	***F***	R^2	**adj. *p***
SF0d vs SF3d	1	1.29	0.18	0.310	8.12	0.58	0.055
SF0d vs SF6d	1	1.76	0.23	0.035	23.25	0.79	0.055
SF0d vs SF9d	1	3.58	0.37	0.320	23.54	0.80	0.055
SF0d vs SF12d	1	2.47	0.29	0.035	23.57	0.80	0.055
SF0d vs SF15d	1	5.98	0.50	0.035	23.57	0.80	0.055
SF3d vs SF6d	1	2.23	0.27	0.035	21.76	0.78	0.055
SF3d vs SF9d	1	5.00	0.45	0.035	22.51	0.79	0.055
SF3d vs SF12d	1	3.50	0.37	0.035	22.50	0.79	0.055
SF3d vs SF15d	1	8.84	0.60	0.035	22.55	0.79	0.055
SF6d vs SF9d	1	3.73	0.38	0.035	2.66	0.31	0.479
SF6d vs SF12d	1	2.40	0.29	0.035	2.69	0.31	0.489
SF6d vs SF15d	1	6.36	0.51	0.035	2.77	0.32	0.240
SF9d vs SF12d	1	2.11	0.26	0.035	0.57	0.09	0.831
SF9d vs SF15d	1	2.98	0.33	0.035	1.12	0.16	0.489
SF12d vs SF15d	1	2.60	0.30	0.035	1.62	0.21	0.175

Presented are degrees of freedom, *F*-values, R^2-values, adj. *p* values from PERMANOVA tests. In the fungal community analysis, several pairwise comparisons (e.g., SF0d vs SF6d) exhibited exceptionally high explanatory power ($R^2 > 0.78$) but yielded marginally significant adjusted *p* values (adj. $p = 0.055$). This is a recognized mathematical artifact caused by the permutation limit in small sample sizes (n = 4). The constrained number of possible permutations caps the minimum attainable raw *p*-value, which, after strict FDR multiple-testing correction, cannot mathematically drop below

the 0.05 threshold. Therefore, the profound community shifts in these stages are strongly supported by the high R^2 values.
